# Simultaneous Determination of Pyridate, Quizalofop-ethyl, and Cyhalofop-butyl Residues in Agricultural Products Using Liquid Chromatography-Tandem Mass Spectrometry

**DOI:** 10.3390/foods11070899

**Published:** 2022-03-22

**Authors:** Jae-Han Shim, Md. Musfiqur Rahman, Ahmed A. Zaky, Shin-Jee Lee, Ara Jo, Seung-Hee Yun, Jong-Bang Eun, Jong-Hwan Kim, Jong-Woo Park, Emel Oz, Charalampos Proestos, Fatih Oz, A. M. Abd El-Aty

**Affiliations:** 1 Natural Products Chemistry Laboratory, Biotechnology Research Institute, Chonnam National University, 77 Yongbong-ro, Buk-gu, Gwangju 61186, Korea; musfiq_707@yahoo.com (M.M.R.); tlswl3763@naver.com (S.-J.L.); Korjapara@daum.net (A.J.); Yun720203@gamail.com (S.-H.Y.); 2 Department of Food Technology, Food Industries and Nutrition Research Institute, National Research Centre, Dokki, Cairo 12622, Egypt; dr.a.alaaeldin2012@gmail.com; 3 Department of Food Science and Technology, College of Agriculture and Life Sciences, Chonnam National University, Gwangju 61186, Korea; jbeun@jnu.ac.kr; 4 Environmental Chemistry Research Group, Korea Institute of Toxicology, 17 Jegok-gil, Munsan-eup, Jinju 52834, Korea; jjong@kitox.re.kr; 5 Analysis Technology and Tomorrow (ATNT), #1105 (Dongwon Biz Platform), 329, Seongseo-ro, Dalseo-gu, Daegu 42703, Korea; jwpark@atnt.co.kr; 6 Department of Food Engineering, Faculty of Agriculture, Ataturk University, Erzurum 25240, Turkey; emel.oz@atauni.edu.tr (E.O.); fatihoz@atauni.edu.tr (F.O.); 7 Laboratory of Food Chemistry, Department of Chemistry, National and Kapodistrian University of Athens Zografou, 15771 Athens, Greece; harpro@chem.uoa.gr; 8 Department of Pharmacology, Faculty of Veterinary Medicine, Cairo University, Giza 12211, Egypt; 9 Department of Medical Pharmacology, Medical Faculty, Ataturk University, Erzurum 25240, Turkey

**Keywords:** pyridate, quizalofop-ethyl, cyhalofop-butyl, Z-SEP, agricultural products, tandem mass spectrometry analysis

## Abstract

An analytical method was developed to simultaneously determine pyridate, quizalofop-ethyl, and cyhalofop-butyl in brown rice, soybean, potato, pepper, and mandarin using LC-MS/MS. Purification was optimized using various sorbents: primary–secondary amine, octadecyl (C18) silica gel, graphitized carbon black, zirconium dioxide-modified silica particles, zirconium dioxide-modified silica particles (Z-SEP), and multi-walled carbon nanotubes (MWCNTs). Three versions of QuECHERS methods were then tested using the optimal purification agent. Finally, samples were extracted using acetonitrile and QuEChERS EN salts and purified using the Z-SEP sorbent. A six-point matrix-matched external calibration curve was constructed for the analytes. Good linearity was achieved with a determination coefficient ≥0.999. The limits of detection and quantification were 0.0075 mg/kg and 0.01 mg/kg, respectively. The method was validated after fortifying the target standards to the blank matrices at three concentration levels with five replicates for each concentration. The average recovery was within an acceptable range (70–120%), with a relative standard deviation <20%. The applicability of the developed method was evaluated with real-world market samples, all of which tested negative for these three herbicide residues. Therefore, this method can be used for the routine analysis of pyridate, quizalofop-ethyl, and cyhalofop-butyl in agricultural products.

## 1. Introduction

Various types of pesticides are being increasingly used worldwide to enhance the production and quality of agricultural commodities and to protect them from pests, diseases, and weeds [1]. Herbicides, in particular, have been an essential tool for practical farming for many years, as they can stop unwanted plant growth while leaving the desired crop unharmed [2]. Hence, herbicides have been widely used in row-crop farming and applied before or during planting [3]. They also may be applied to crops in the fall to improve harvesting [4]. Despite these benefits, herbicides inevitably cause serious environmental and health concerns [5]. This is because many agricultural products are left with residues of various herbicides, which naturally poses a potentially hazardous risk to consumers and various ecosystems [6]. Thus, herbicide residues in agricultural commodities should be analyzed to improve public health and food safety [7].

The Positive List System (PLS) was recently implemented to strengthen pesticide acceptance standards for safe agricultural products in the Republic of Korea; as a result, quantitative analysis methods should have detection levels as low as 0.01 mg/kg. Thus, more practical strategies are urgently required to monitor various xenobiotics in different foods (e.g., cereals, root and tuber crops, legumes, fruits, and vegetables). The Ministry of Food and Drug Safety (MFDS) has been developing simultaneous methods to meet the requirement for the residue analysis of pesticides in agricultural foods using liquid chromatography-tandem mass spectrometry (LC-MS/MS) [8]. Notably, pyridate, quizalofop-ethyl, and cyhalofop-butyl residues could not be determined, which necessitated the development of a method that could detect these compounds.

Cyhalofop-butyl and quizalofop-ethyl, which belong to a group of phenoxy compounds, have remarkable crop selectivity, are highly effective in controlling weeds, and demonstrate good systemic abilities [9]. The mechanism of action of these compounds is based on the inhibition of acetyl CoA carboxylase (ACCase); this excludes pyridate, which functions as a photosynthetic electron transport inhibitor [10]. Pyridate controls annual broad-leaved weeds, especially triazine-resistant biotypes and some grass weeds [11]. Quizalofop-ethyl, introduced by Nissan Chemical Industries Ltd. in 1984, was used for the selective post-emergence control of annual and perennial grass weeds in potatoes, soybeans, peanuts, and cotton [12]. Cyhalofop-butyl was manufactured in the mid-1980s by The Dow Chemical Company (now Dow AgroSciences). It is used to control barnyard grass in rice paddies [13]. At present, pyridate is not used domestically. However, the analysis of cyhalofop-butyl residues in agricultural products needs to be further developed because several countries that export agricultural products to Korea use this herbicide. 

Currently, there are a few literature methods on the analyses of these herbicides (pyridate, quizalofop-ethyl, and cyhalofop-butyl) residues. For example, a study by Wu et al. [13] identified cyhalofop-butyl and its metabolite in a rice ecosystem using high-performance LC liquid chromatography (HPLC)-MS/MS. This technique was also employed by Lindner et al. [11] to detect pyridate in cereals. Furthermore, quizalofop-ethyl has been detected in soils and plants by gas-liquid chromatography (GLC) and enzyme-linked immunosorbent assays (ELISAs) [14]. However, no existing technology can analyze these herbicide residues simultaneously. Additionally, the methods mentioned above are time-consuming and environmentally harmful. The simultaneous determination of pesticides is a desirable and practical approach because it would provide increased efficiency, lower costs, and reduced labor relative to conventional methods [15,16]. Moreover, it would reduce the amount of materials required for experiments, thus protecting the environment. However, simultaneous analysis requires the removal of interferences (e.g., matrix components) and the consideration of analyte characteristics [17]. 

Presently, the quick, easy, cheap, rugged, effective, and safe (“QuEChERS”) sample preparation method is earning popularity in the area of green chemistry because it enables multi-residue pesticide analysis for complex matrices and minimizes sample size and the quantity of required materials [18,19,20]. It can be applied to pesticides with diverse polarity ranges and volatility [21]. LC-MS/MS is another powerful tool for analyzing pesticide residues in complex matrices [22]. It is characterized by high precision and sensitivity with fewer extraction procedures than other methodologies, such as gas chromatography-mass spectrometry (GC-MS) [23]. Unlike GC analysis, sample volatilization is not required for LC, which avoids problems associated with chemical degradation and the formation of new products common under high heat conditions. Further, LC–MS/MS specimens typically require no derivatization and minimal sample preparation. In some cases, the specimens can be diluted and directly injected into the LC-MS/MS, significantly increasing throughput. Consequently, the present study aimed to establish an accurate, cost-effective, and environmentally friendly simultaneous detection method for detecting herbicides (pyridate, cyhalofop-butyl, and quizalofop-ethyl) in different agricultural products (brown rice, soybean, potato, pepper, and mandarin.) Because the complex matrices of farming products affect analysis precision, this study employs sample extraction and clean-up (pre-treatment) steps [24]. Specifically, samples were extracted by QuEChERS methods (Original, AOAC, and EN), followed by clean-up and analysis using LC-M/MS.

## 2. Materials and Methods

### 2.1. Chemicals and Reagents 

Cyhalofop-butyl (99.7%), quizalofop-ethyl (96.4%), and pyridate (96%) were procured from Wako (Richmond, VA, USA), Sigma–Aldrich (Buchs, Switzerland), and Dr. Ehrenstorfer (Augsburg, Germany), respectively. The physicochemical characteristics are shown in Table 1. Acetonitrile and methanol (HPLC grade) were obtained from J.T. Baker (Avantor, Radnor, PA, USA). Formic acid was acquired from Daejung (Siheung-si, Korea), ammonium formate was secured from Sigma–Aldrich and QuEChERS kits (Original, EN, AOAC) were supplied by KRIAT (Daejeon, Korea). Zirconium dioxide-modified silica particles (Z-SEP) and zirconium dioxide-modified silica particles + (Z-SEP+) were obtained from Supelco (Bellefonte, PA, USA). Primary–secondary amine (PSA), octadecyl (C18) silica gel, and graphitized carbon black (GCB) sorbents were acquired from Agilent (Santa Clara, CA, USA). Multi-walled carbon nanotubes (MWCNTs) were supplied by Cheap Tubes (Grafton Vermont, VT, USA). Matrices used for establishing the residual-detection method include cereal (brown rice), legume (soybean), root and tuber crops (potato), vegetables (pepper), and fruits (mandarin); they were homogenized and stored in a sealed container at −70 °C.

### 2.2. Preparation of Standard Stock Solution and Calibration

Solutions of pyridate, quizalofop-ethyl, and cyhalofop-butyl in methanol (1000 ppm) were separately prepared. The stock solution was diluted with the same solvent to 100 ppm and used as a mixed standard solution. To prepare a calibration curve, the intermediate standards solutions of the three pesticides were diluted to 2.5, 5, 7.5, 10, 25, and 50 ng/mL. The standard stock, intermediate, and working solutions were stored at 4 °C until analysis. Brown vials were used throughout the experiment.

### 2.3. Sample Preparation 

A homogenized sample (10 g, 5 g of both cereal and legume) was placed in a 50-mL Teflon centrifuge tube. A 20 mL acetonitrile (for grain and legume, 5 mL of water was added, wet for 30 min, and 10 mL of acetonitrile was added) was then added to the sample in the centrifuge tube, and the mixture was homogenized with a tissue homogenizer for 1 min. A QuEChERS EN extraction kit containing 4 g of MgSO_4_, 1 g of NaCl, 1 g of trisodium citrate dihydrate, and 0.5 g of disodium hydrogencitrate sesquihydrate was shaken manually for 1 min. The sample tube was centrifuged at 4000 rpm for 5 min, and a 1.5-mL portion of the supernatant was transferred to a microtube that contained 75 mg of Z-SEP. The microtube was then vortexed for 1 min and centrifuged at 4000 rpm for 5 min. The supernatant was diluted with methanol (1:1) and then transferred to a vial for LC-MS/MS analysis.

### 2.4. Instrumental Conditions

An Alliance 2695 LC separation module (Waters, Milford, MA, USA) coupled with a Micromass Quattro Micro triple quadrupole tandem mass spectrometer (Waters) was used to detect and analyze pyridate, quizalofop-butyl, and cyhalofop-ethyl in five food products (brown rice, soybean, pepper, potato, and mandarin). An Agilent ZORBAX Eclipse Plus C18 column (3.0 mm I.D × 150 mm, 3.5 μm) was used for separation (Table 2).

The MS/MS was operated in multiple reaction monitoring (MRM) mode with positive electrospray ionization. MassLynx (version 4.1) software was used for data acquisition.

### 2.5. Statistical Analyses

The statistics required for interpreting analytical method validation results are the calculation of the mean, standard deviation, relative standard deviation, and regression analysis (IBM SPSS Statistics, v25, Armonk, NY, USA). The acceptance criteria for each validation characteristic are typically around the individual values as well as the mean and relative standard deviation.

## 3. Results and Discussion

### 3.1. Optimization of the Extraction Procedure

The extraction conditions were first considered by referring to the physicochemical properties of the three analytes (pyridate, cyhalofop-butyl, and quizalofop-ethyl). When a sample contains moisture, its surface is hydrated with water; this provides better extraction efficiency when using water-soluble organic solvents than non-polar organic solvents with low permeability. Typical water-soluble organic solvents include acetonitrile and acetone, although acetonitrile is preferentially considered because acetone had the disadvantage of extracting large amounts of non-polar or polar interference materials. To simplify the pre-processing of the samples, we employed the QuEChERS method, which has advantages of relatively low solvent usage and rapid analysis time [25]. According to the reagents used, the QuEChERS method is divided into three categories: original, EN, and AOAC. A comparison of these extraction methods showed differences in their average recovery rates. Among the grain, root and tuber crop, legume, fruit, and vegetable matrices, the original method could not recover the three analytes in an average of 70% to 120% from three matrices. In contrast, the AOAC method could not recover 70% to 120% of the analytes from all six matrices. Therefore, with the best average recovery rate for all samples, the EN method was selected and used for the experiments (Table 3).

### 3.2. Optimization of Clean-Up Procedure

Typical clean-up procedures are not always satisfactory for analysis, especially for pigments, sugars, and organic acids [26]. Thus, we considered various purification agents to develop a new clean-up method for analyzing our target herbicide residues. The optimal sorbent was selected by comparing the recovery rates of the analytes obtained using different quantities of sorbent. First, C18 and PSA, which effectively remove organic acids, sugars, oil, and fat components, were considered. GCB and MWCNTs, which can effectively remove pigments, such as sterol, chlorophyll, and carotenoid, were also tested [27]. When 50, 75, or 100 mg of C18 was used in the clean-up step, there was a decline in the recovery value of pyridate (14.3%, 10.0%, and 1.8%, respectively). Similarly, with 50, 75, or 100 mg of PSA, the recovery value of the three tested analytes was remarkably low (0%). Notably, 50, 75, and 100 mg of GCB were adequate for recovering cyhalofop-butyl (88–108%), but there were slight decreases in the recovery value of quizalofop-ethyl (7–50%) and pyridate (10–32%). Furthermore, the efficiency of the MWCNTs sorbent was excellent for cyhalofop-butyl recovery (58–107%) but not for those of quizalofop-ethyl (0–1.6%) and pyridate (0–5.8%). Based on these results, we employed Z-SEP and Z-SEP+ as alternative absorbents for removing pigments. These are relatively new, commercially available dispersive phases that can effectively remove larger amounts of fat and coloring from samples than conventional sorbents like C18 and PSA [27]. With Z-SEP and Z-SEP+, the recovery value of quizalofop-ethyl and pyridate was 77–220%. However, Z-SEP provided a better recovery value for cyhalofop-butyl (71–99%) than Z-SEP+ (105–139%). Finally, Z-SEP (75 mg) was chosen as the optimal sorbent due to its reasonable recovery rates; this excluded recovery rates of less than 70% and more than 120% (Table 4).

### 3.3. Analytical Procedure

The positive electrospray ionization (ESI) mode was used to detect pyridate, cyhalofop-butyl, and quizalofop-butyl in agricultural products. One ppm of each standard solution was injected into the mass detector, the production ion was selected through the production scan mode, and collision energy with high detection intensity was selected. The product ions detected with the best sensitivity were considered quantitative ions, while those detected with poorer sensitivity were considered qualitative ions. The characteristic ions chosen for analysis and optimal collision energy are shown in Table 5. It is worth noting that because LC-MS/MS analysis can suppress ionization or augment target components due to non-target components extracted from the sample, we quantified the analytes based on matrix-matched calibration (MMC).

### 3.4. Method Validation

#### 3.4.1. Specificity

The selectivities of pyridate, cyhalofop-butyl, and quizalofop-ethyl were evaluated by comparing the blank and spiked samples’ chromatograms to those of the standard solutions. An analysis of the amounts of herbicides recovered from the spiked samples compared to the blank samples showed that this method has high separation and selectivity for analyzing pyridate, cyhalofop-butyl, and quizalofop-ethyl in agricultural products. No interference was detected with the same retention time mass-to-charge ratio (*m*/*z*).

#### 3.4.2. Matrix Effects and Linearity

To evaluate and minimize matrix effects on the analyte responses and performances, the comparative data between solvent calibration curves were used following the Kanrar equation [28].

$$\mathrm{Matrix}\;\mathrm{effect}\;\left(\%\right)\;=\frac{\mathrm{Standard}\;\mathrm{in}\;\mathrm{matrix}\;\mathrm{peak}\;\mathrm{area}\;-\;\mathrm{standard}\;\mathrm{in}\;\mathrm{solvent}\;\mathrm{peak}\;\mathrm{area}}{\mathrm{Standard}\;\mathrm{in}\;\mathrm{solvent}\;\mathrm{peak}\;\mathrm{area}}$$

To check the linearity of the matrix-matched standard solution, a mixed stock solution comprising all three analytes was diluted with the blank sample of each extract (brown rice, soybean, green pepper, potato, and mandarin). Then 5 µL of various concentrations of these samples (0.0025, 0.005, 0.0075, 0.01, 0.025, or 0.05 µg/mL) were injected into the LC-MS/MS system and analyzed. The linearity was satisfactory with a coefficient of determination (*R*^2^) of ≥0.99.

The ME was tested at a concentration rate of 0.05 ppm, and the value was ranged from +21–+43, −33–+42, and −10–+17 for cyhalofop-butyl, quizalofop-ethyl, and pyridate, respectively. On the other hand, the values were in between −33–+21, +17–+40, +10–+44, +7–+41, and +7–43, in brown rice, soybean, mandarin, pepper, and potato, respectively. As MEs cannot be ruled out, matrix-matched calibration was used throughout the experimental work and quantification. 

#### 3.4.3. Limits of Detection and Quantitation

The LODs and LOQs of pyridate, quizalofop-ethyl, and cyhalofop-butyl were calculated using the test solution preparation and device analysis methods. The calculated LOD and LOQ were obtained as signal-to-noise ratios of (S/N) ≥3 and S/N ≥10. The LOD and LOQ were 0.0075 mg/kg and 0.01 mg/kg, respectively, for all matrices. For comparison, a previous work detecting cyhalofop-butyl in a rice paddy field achieved a LOQ of 0.001 mg/kg and a LOD of 0.003 mg/kg [13].

#### 3.4.4. Recovery 

**Figure 1:**
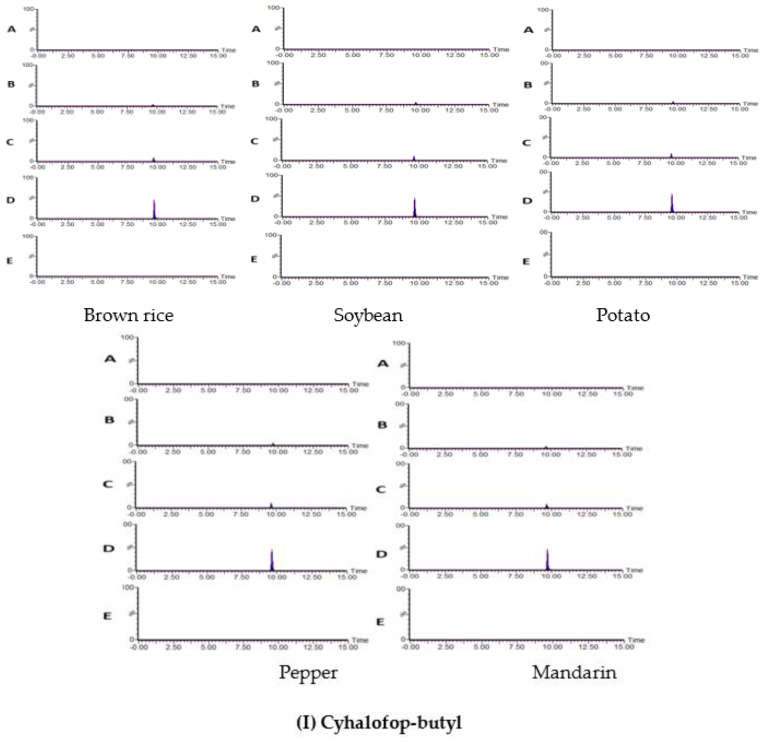

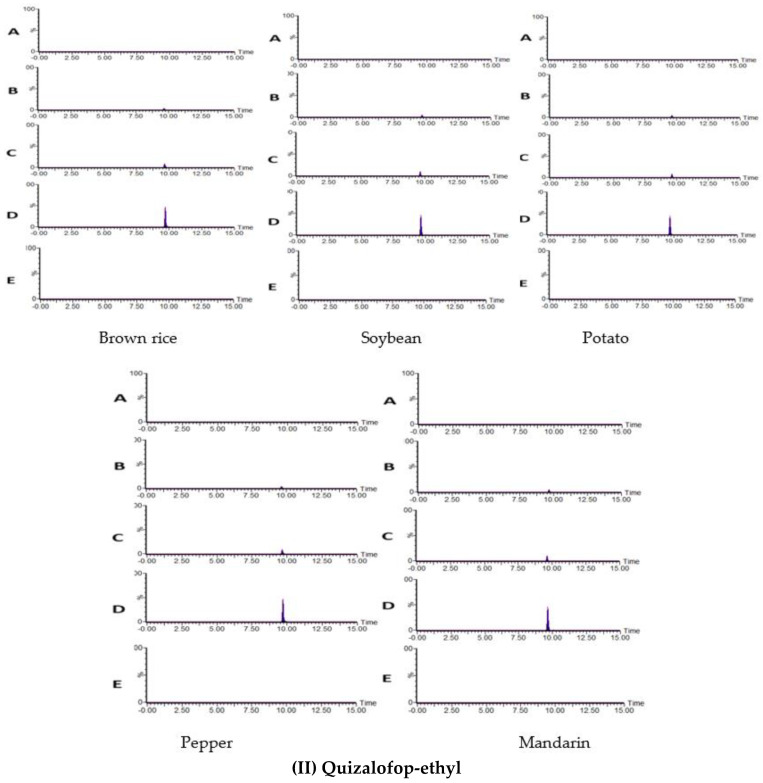

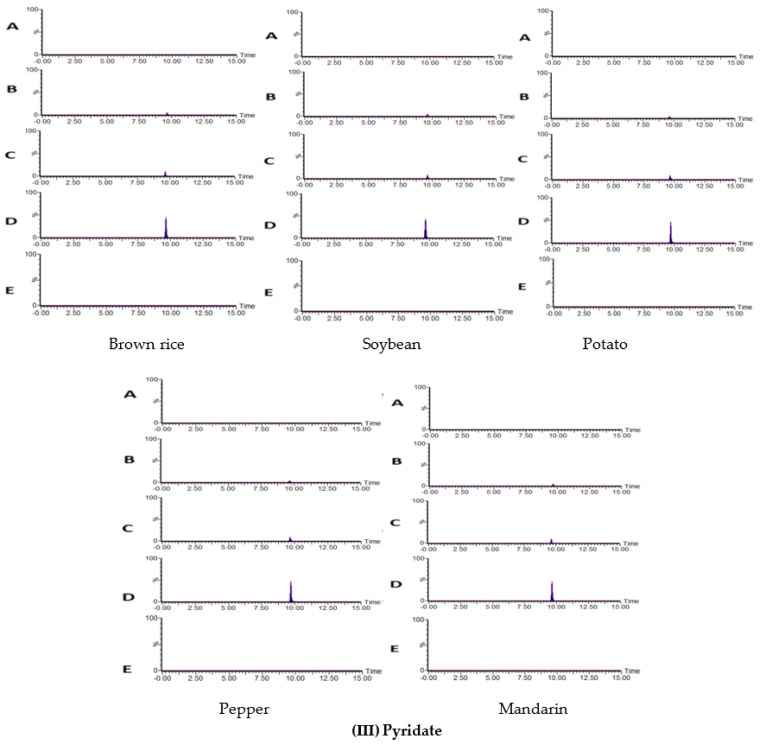
Representative MRM (quantification ion) chromatograms of (**I**) cyhalofop-butyl, (**II**) quizalofop-ethyl, and (**III**) pyridate in five different matrices (brown rice, soybean, potato, pepper, and mandarin: (**A**) blank sample, (**B**) sample spiked at 0.005 mg/kg, (**C**) sample spiked at 0.01 mg/kg, (**D**) sample spiked at 0.2 mg/kg, and (**E**) market sample.

To evaluate the accuracy, reproducibility, and efficiency of our method, experiments for recovery of pyridate, quizalofop-ethyl, and cyhalofop-butyl were conducted five times with fortification levels of 0.01, 0.02, and 0.1 mg/kg, corresponding to the following levels: LOQ, 2 × LOQ, and 10 × LOQ. The average recovery of pyridate, quizalofop-ethyl, and cyhalofop-butyl are as follows: pyridate 85.7–109.6%; quizalofop-ethyl 89.1–112.4%; and cyhalofop-butyl 84.5–106.5%. The respective RSD values were 9.2%, 8.9%, and 15.0%. It was confirmed that these values meet the standards of the codex guidelines (recovery rate = 70–120%) [29] and the Korea Food and Drug Safety Assessment Institute’s “Guidelines on Standard Procedures for Preparation of Test Methods (RSDs < 22%) [30]” regarding the analysis of pesticide residues (Table 6). Cross-validation was carried out for this established test method at two verification institutions, which determined a recovery value of 74.3–117.6% for each concentration of all samples; the standard deviation was within 15% (Table 7). Consequently, compliance with the test method verification criteria was confirmed. Recovery chromatograms of pyridate, cyhalofop-butyl, and quizalofop-ethyl are shown in Figure 1. Moreover, of note is the recovery value (78.9–107.5%, standard deviation = 2.0–6.9%) of cyhalofop-butyl obtained when using HPLC-MS/MS with C18 as an adsorbent [13], which were lower than those achieved by our LC-MS/MS methods using Z-SEP as the adsorbent.

### 3.5. Method Application

The developed method was applied to detect pyridate, cyhalofop-butyl, and quizalofop-ethyl in soybean, mandarin, pepper, potato, and brown rice (10 per each matrix) procured from different local markets in Gwangju, Republic of Korea. The samples were extracted and analyzed according to the above-established procedure. None of the samples showed any residues of pyridate, cyhalofop-butyl, or quizalofop-ethyl (Figure 1). In line with our finding, Wu et al. [13] found that the ultimate residues of cyhalofop-butyl and its metabolite in the rice samples were not detectable or below 0.01 mg/kg at harvest.

## 4. Conclusions

A simple LC-MS/MS method was developed and optimized for the simultaneous analysis of pyridate, quizalofop-butyl, and cyhalofop-ethyl in five agricultural products (soybean, mandarin, pepper, potato, and brown rice). Among the three types of QuEChERS methods, the EN version most effectively extracted the target analytes. Furthermore, Z-SEP demonstrated a superior analyte recovery rate to those of the other evaluated sorbents (C18, GCB, PSA, MWCNTs, and Z-SEP+). These components provided a system capable of multi-residue detection within complex matrices, with sample sizes and quantities of required materials lower than those of conventional detection methods. The recovery and repeatability of the developed method are within the acceptable range as determined by CODEX guidelines, thus making our LC-MS/MS system suitable for monitoring pyridate, quizalofop-ethyl, and cyhalofop-butyl residues in agricultural products. Shandong.

## Figures and Tables

**Table 1:** Physicochemical characteristics of pyridate, cyhalofop-butyl, and quizalofop-ethyl.

Analyte	CAS No.	Molecular Formula	Chemical Structure	Log P_ow_
Cyhalofop-butyl	122008-85-9	C_20_H_20_FNO_4_	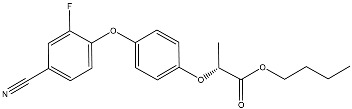	3.31
Quizalofop-ethyl	76578-14-8	C_19_H_17_ClN_2_O_4_	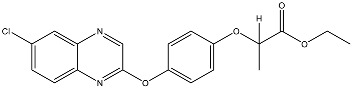	4.28
Pyridate	55512-33-9	C_19_H_23_ClN_2_O_2_S	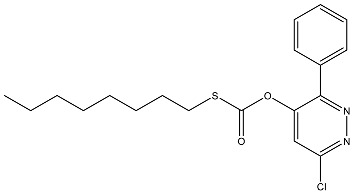	6.61

**Table 2:** Analytical conditions for the determination of the tested analytes in various matrices.

Condition	Content		
Instrument	LC: Alience 2695 LC separation module, Waters MS/MS: Micromass Quattro Micro triple quadrupole tandem mass, Waters		
Chromatographic separation			
Column	Agilent ZORBAX Eclipse Plus C18 (3.0 mm I.D. × 150 mm L, 3.5 μm)		
Flow rate	0.3 mL/min		
Injection volume	5 μL		
Oven temp.	40 °C		
Mobile phase	A: 5 mM ammonium formate, 0.1% formic acid in methanol B: 5 mM ammonium formate, 0.1% formic acid in water		
Gradient	Time	A (%)	B (%)
	0.0	5	95
	2.0	5	95
	4.0	95	5
	10.0	95	5
	12.0	5	95
	15.0	5	95
MS/MS condition			
Capillary voltage	3.9 kV		
Source temp.	150 °C		
Desolvation gas temp.	250 °C		
Desolvation gas flow	500 L/h		

**Table 3:** Comparison of QuEChERS extractions for the determination of three residual herbicides in (A) brown rice, (B) soybean, (C) potato, (D) pepper, and (E) mandarin matrices.

Analyte		Spiking Level	Recovery ± RSD ^a^ (%)		
			Original	AOAC	EN
(A)	Cyhalofop-butyl	LOQ	110.6 ± 8.4	71.2 ± 22.5	98.3 ± 4.2
		10 × LOQ	86.3 ± 7.7	127.3 ± 12.7	101.5 ± 1.5
	Quizalofop-ethyl	LOQ	76.8 ± 0.6	111.0 ± 7.6	96.5 ± 7.2
		10 × LOQ	99.0 ± 6.2	104.2 ± 0.6	94.4 ± 3.6
	Pyridate	LOQ	92.2 ± 7.9	121.9 ± 8.5	82.0 ± 8.7
		10 × LOQ	91.0 ± 4.3	86.8 ± 9.2	81.3 ± 1.1
(B)	Cyhalofop-butyl	LOQ	51.3 ± 19.9	72.3 ± 22.0	80.0 ± 12.2
		10 × LOQ	78.6 ± 2.2	101.5 ± 17.6	95.4 ± 12.5
	Quizalofop-ethyl	LOQ	85.5 ± 3.3	99.3 ± 2.7	86.3 ± 1.7
		10 × LOQ	85.2 ± 1.3	108.5 ± 7.9	102.4 ± 1.9
	Pyridate	LOQ	87.9 ± 0.4	85.5 ± 3.5	78.0 ± 4.5
		10 × LOQ	77.0 ± 3.5	86.8 ± 11.0	83.6 ± 0.7
(C)	Cyhalofop-butyl	LOQ	93.9 ± 1.7	115.3 ± 0.3	76.5 ± 9.2
		10 × LOQ	113.5 ± 6.9	124.6 ± 9.4	102.4 ± 13.2
	Quizalofop-ethyl	LOQ	90.5 ± 4.6	129.3 ± 1.0	104.2 ± 1.7
		10 × LOQ	92.4 ± 7.5	126.3 ± 8.5	106.9 ± 5.5
	Pyridate	LOQ	76.9 ± 2.0	128.1 ± 3.3	86.7 ± 10.4
		10 × LOQ	92.8 ± 6.5	110.5 ± 0.6	99.9 ± 1.7
(D)	Cyhalofop-butyl	LOQ	81.5 ± 37.7	27.8 ± 11.4	86.5 ± 2.6
		10 × LOQ	62.7 ± 14.1	100.9 ± 7.7	85.1 ± 4.9
	Quizalofop-ethyl	LOQ	68.4 ± 7.2	90.0 ± 6.9	93.3 ± 5.0
		10 × LOQ	75.9 ± 1.1	92.4 ± 3.3	91.7 ± 0.7
	Pyridate	LOQ	80.2 ± 11.2	87.3 ± 11.2	100.5 ± 1.9
		10 × LOQ	76.9 ± 3.4	97.0 ± 2.8	90.3 ± 4.8
(E)	Cyhalofop-butyl	LOQ	117.5 ± 14.0	95.8 ± 35.0	80.4 ± 17.3
		10 × LOQ	75.4 ± 0.8	91.0 ± 6.9	88.7 ± 9.0
	Quizalofop-ethyl	LOQ	81.1 ± 5.0	99.0 ± 4.6	82.2 ± 5.7
		10 × LOQ	94.0 ± 1.2	101.7 ± 7.0	94.0 ± 0.4
	Pyridate	LOQ	70.1 ± 7.1	78.4 ± 7.8	79.8 ± 5.2
		10 × LOQ	84.1 ± 1.2	81.2 ± 1.9	73.2 ± 2.1

^a^ Mean value of three measurements with relative standard deviation.

**Table 4:** Optimization of sorbent type and amounts in terms of analyte recovery.

Purification Material		Recovery (%)		
		Cyhalofop-butyl	Quizalofop-ethyl	Pyridate
PSA	50 mg	121.4	89.8	0.0
	75 mg	70.0	96.4	0.0
	100 mg	104.6	82.2	0.0
C_18_	50 mg	107.4	90.2	14.3
	75 mg	98.3	87.4	10.0
	100 mg	94.6	74.0	1.8
GCB	5 mg	108.0	50.2	32.6
	10 mg	95.9	20.7	14.4
	15 mg	88.4	6.9	10.0
MWCNTs	5 mg	107.2	1.6	5.8
	10 mg	58.4	0.0	2.1
	15 mg	57.9	0.0	0.0
Z-SEP	50 mg	92.2	88.3	110.3
	75 mg	99.5	101.4	93.2
	100 mg	71.3	91.3	+95.9
Z-SEP+	50 mg	105.1	86.2	77.2
	75 mg	139.7	101.1	98.3
	100 mg	124.0	90.1	81.9

Mean value of four measurements.

**Table 5:** Characteristic ions observed via LC-MS/MS for three residual herbicides.

Analyte		Molecular Weight (g/mol)	Exact Mass (g/mol)	Precursor Ion (*m*/*z*)	Product Ion (*m*/*z*)	CE ^a^ (V)
1	Cyhalofop-butyl	357.4	357.14	358.1319	120.0795	26
					256.1212 ^b^	10
2	Quizalofop-ethyl	372.8	372.09	373.0561	91.062	32
					299.1621 ^b^	20
3	Pyridate	378.9	378.12	379.1319	207.0993 ^b^	28
					351.1753	10

^a^ Collision energy; ^b^ quantitative ion.

**Table 6:** Validation results of the proposed analytical method for the determination of three residual herbicides in food samples.

Analyte	Spiking Level (mg/kg)	Recovery ± RSD ^a^ (%)					LOQ (mg/kg)
		Brown Rice	Soybean	Potato	Green Pepper	Mandarin	
Cyhalofop-butyl	0.01	87.0 ± 15.0	85.9 ± 14.8	102.9 ± 6.8	84.5 ± 4.5	92.0 ± 12.7	0.01
	0.02	91.1 ± 9.8	95.6 ± 8.2	92.8 ± 10.0	93.4 ± 7.0	105.8 ± 9.3	
	0.1	96.9 ± 8.2	106.5 ± 6.0	89.8 ± 4.5	90.3 ± 7.1	94.4 ± 7.9	
Quizalofop-ethyl	0.01	94.4 ± 7.8	101.3 ± 7.4	97.5 ± 3.8	89.1 ± 8.5	98.5 ± 6.9	0.01
	0.02	97.8 ± 6.6	104.4 ± 3.1	91.2 ± 5.5	99.4 ± 6.7	104.7 ± 8.9	
	0.1	91.2 ± 5.2	106.0 ± 1.5	92.0 ± 1.8	103.8 ± 1.8	112.4 ± 4.0	
Pyridate	0.01	106.8 ± 2.9	94.6 ± 5.6	91.0 ± 3.0	89.9 ± 5.4	93.6 ± 3.4	0.01
	0.02	99.6 ± 2.0	90.6 ± 4.2	94.2 ± 5.5	86.8 ± 2.8	91.8 ± 9.2	
	0.1	109.6 ± 3.7	88.4 ± 3.1	94.3 ± 2.6	106.4 ± 0.5	85.7 ± 3.7	

^a^ Mean value of five measurements with relative standard deviation.

**Table 7:** Inter-lab A and B validation results of the proposed analytical method for the determination of three residual herbicides in food samples.

Analyte	Spiking Level (mg/kg)		Recovery ± RSD ^a^ (%)				
			Brown Rice	Soybean	Potato	Green Pepper	Mandarin
Cyhalofop-butyl	0.01	A	105.6 ± 8.3	117.6 ± 4.6	84.9 ± 3.5	99.6 ± 4.1	75.8 ± 9.9
		B	87.0 ± 2.8	95.0 ± 1.8	95.3 ± 2.2	94.0 ± 4.1	95.3 ± 4.7
	0.02	A	103.4 ± 6.9	104.3 ± 4.9	91.3 ± 6.2	82.0 ± 8.7	79.4 ± 8.0
		B	88.2 ± 3.0	93.2 ± 5.1	86.2 ± 3.4	92.4 ± 1.6	98.3 ± 3.7
	0.1	A	102.5 ± 5.5	90.5 ± 6.0	90.9 ± 4.5	79.8 ± 3.5	96.8 ± 7.8
		B	86.9 ± 3.6	103.0 ± 2.3	83.7 ± 2.5	93.0 ± 3.1	99.2 ± 1.7
Quizalofop-ethyl	0.01	A	71.8 ± 2.7	88.9 ± 2.0	72.2 ± 7.5	76.7 ± 9.9	76.6 ± 4.4
		B	81.3 ± 3.2	83.7 ± 5.5	89.5 ± 3.2	101.0 ± 2.9	112.9 ± 6.5
	0.02	A	81.3 ± 4.6	81.6 ± 2.0	80.2 ± 3.3	89.6 ± 2.4	83.7 ± 2.1
		B	86.9 ± 1.5	85.4 ± 1.9	91.9 ± 3.4	96.5 ± 1.0	111.1 ± 4.2
	0.1	A	78.2 ± 5.4	74.9 ± 1.4	82.4 ± 1.7	97.3 ± 2.2	98.8 ± 3.5
		B	85.6 ± 4.6	94.0 ± 8.3	99.8 ± 4.1	88.7 ± 4.1	105.9 ± 1.8
Pyridate	0.01	A	89.6 ± 3.2	107.1 ± 2.9	86.3 ± 6.2	74.3 ± 3.3	74.5 ± 4.8
		B	85.5 ± 2.2	92.7 ± 3.4	89.1 ± 3.1	103.4 ± 2.9	98.3 ± 1.5
	0.02	A	86.9 ± 5.1	105.6 ± 2.9	82.2 ± 1.9	86.9 ± 5.1	105.6 ± 2.9
		B	86.1 ± 1.8	91.4 ± 0.8	93.7 ± 5.4	99.5 ± 1.5	99.0 ± 1.0
	0.1	A	80.2 ± 3.9	97.8 ± 1.6	97.5 ± 3.4	80.2 ± 3.9	97.8 ± 1.6
		B	87.5 ± 2.2	95.0 ± 1.7	97.2 ± 3.1	93.8 ± 1.5	98.9 ± 1.0

^a^ Mean value of five measurements with relative standard deviation.

## Data Availability

The data presented in this study are available on request from the corresponding author.
